# When Cancer Research Met Artificial Intelligence: From Machine Learning to Intelligent Oncology

**DOI:** 10.3390/cancers18172896

**Published:** 2026-09-07

**Authors:** Daniele Giansanti

**Affiliations:** Centro IATIS, Istituto Superiore di Sanità, 00161 Roma, Italy; daniele.giansanti@iss.it

Cancer research and artificial intelligence have developed from distinct scientific traditions, yet their trajectories are increasingly converging. Cancer research has evolved into a highly multidisciplinary field encompassing tumor biology, diagnosis, prognosis, treatment, prevention, and patient care, while AI has progressed through increasingly sophisticated approaches to data analysis, pattern recognition, prediction, and decision-making. Their intersection is therefore not the result of a single technological breakthrough, but of a gradual integration of computational intelligence across oncology.

This convergence has accelerated in recent years, with AI increasingly explored across the cancer continuum, from biomedical research and early detection to medical imaging, digital pathology, molecular profiling, prognosis, treatment, and patient care. At the same time, machine learning, deep learning, generative AI, foundation models, multimodal systems, and emerging approaches such as digital twins are creating new opportunities to integrate heterogeneous biological, clinical, and imaging information.

In this Editorial, we briefly trace the historical and conceptual evolution of the relationship between cancer and AI, identify representative milestones through a qualitative PubMed analysis, and consider selected areas in which AI is shaping contemporary oncology. Finally, we discuss emerging directions that may influence the future of “Cancer Research, Diagnosis, and Care” providing the broader context for the Special Issue *“The Use of Artificial Intelligence in Cancer Research, Diagnosis, and Care.”*

*Defining Cancer, Oncology, and Artificial Intelligence*

Merriam-Webster defines *cancer* as a malignant tumor characterized by potentially unlimited growth, local invasion, and systemic spread through metastasis [1]. The term, however, encompasses a highly heterogeneous group of diseases, differing in biological mechanisms, anatomical sites, clinical behavior, and therapeutic needs. Cancer research and management therefore extend across a broad spectrum, from understanding tumor biology and prevention to detection, diagnosis, prognosis, treatment, monitoring, and care.

*Oncology* is defined as the branch of medicine concerned with the prevention, diagnosis, treatment, and study of cancer [2]. It consequently represents a broad scientific and clinical field in which biological, molecular, imaging, and clinical information increasingly converge. This complexity has created an expanding role for computational methods capable of extracting meaningful information from large and heterogeneous datasets.

Merriam-Webster defines *artificial intelligence (AI)* as “the capability of computer systems or algorithms to imitate intelligent human behavior” [3]. In practice, AI encompasses a wide range of methods that can learn from data, identify patterns, generate information, make predictions, and assist decision-making. Its growing application in oncology reflects the increasing need to integrate diverse forms of information and address complex problems across cancer research, diagnosis, and care.

The meeting between these domains, however, did not begin with the widespread use of the expression *artificial intelligence*. Computational approaches such as automated pattern recognition, classification, expert systems, and neural networks were already being investigated in cancer-related applications. A meaningful historical perspective must therefore consider the broader evolution of computational methods alongside the emergence of AI terminology. This provides the starting point for mapping how the two fields progressively converged.

*Tracing the Historical Convergence of Cancer and Artificial Intelligence*

To explore how cancer and artificial intelligence have progressively intersected, we performed a qualitative examination of the PubMed-indexed literature using Title/Abstract (tiab) searches ([Table 1]). Rather than attempting to reconstruct the complete history of either field or to identify all relevant publications, the search strategy was designed to identify representative records marking different conceptual stages. Five search positions were considered. Position 1 used broad AI-related terminology and approaches; Position 2 focused on the emergence of cancer and oncology-related terminology; Position 3 identified records explicitly using the expression *artificial intelligence*; Position 4 combined cancer/oncology-related terminology with broader AI-related concepts and computational approaches; and Position 5 combined cancer/oncology-related terminology specifically with *artificial intelligence*. The resulting records were therefore considered historical milestones illustrating the progressive development and eventual convergence of the two domains, rather than evidence from a systematic literature review.

An interesting feature of this historical reconstruction is that the five positions do not simply form a chronological sequence. Instead, they represent different conceptual trajectories that subsequently intersect. The broad AI-related search ([Table 1], Position 1) retrieved *The Medical Expert System in Germany*, published in 1879 [4]. The terminology used in this historical record obviously predates the modern meaning of artificial intelligence and should not be interpreted as evidence of AI in its contemporary sense. Nevertheless, the presence of the expression *expert system* is noteworthy when viewed retrospectively, since expert systems subsequently became an important class of AI-based approaches. This record therefore illustrates how terminology associated with later computational intelligence can appear in historical sources before the establishment of AI as a formal scientific field.

The cancer trajectory can be traced considerably further back. The cancer-only search ([Table 1], Position 2) identified *Some Remarks on the Nature and Treatment of Cancers*, published in 1784 [5]. This record represents an early PubMed-indexed occurrence of terminology explicitly related to cancer and provides a historical marker for the development of the cancer domain as a recognizable subject of medical investigation. At this stage, of course, cancer research was entirely situated within the clinical and pathological knowledge of its time, with no relationship to computational methods. The finding is nevertheless useful for establishing the temporal depth of the cancer trajectory against which the much later emergence of AI can be considered.

A distinct trajectory is represented by the development of artificial intelligence as an explicit scientific concept. The AI-only search ([Table 1], Position 3) identified Maron’s *Artificial intelligence and brain mechanisms*, published in 1963 [6]. Its relevance lies principally in the explicit use of the expression *artificial intelligence*. By this period, AI was beginning to emerge as a recognizable area of computational research concerned with the representation and simulation of aspects of intelligent behavior. Thus, by the early 1960s, the cancer field had already developed a substantial historical background, while AI was establishing itself as a distinct computational paradigm. The two trajectories, however, had not yet explicitly converged.

The first clear bridge between cancer-related applications and broad AI-related computational approaches identified by our search appears in 1967. Dawson, Heanley, Heber-Percy, and Tylko reported *Cesar: cervical smear analyser and reader. A new approach to evaluating cells in cytological preparations* [7]. The study described an automated multiparameter approach to pattern recognition for the detection of malignant cells, including cervical and other cancers, with cells being classified through a cybernetic approach. This record is particularly important for Position 4 because it combines a cancer-related application with computational concepts that belong to the broader historical development of AI, without necessarily relying on the modern expression *artificial intelligence*. In this sense, the 1967 contribution represents an early example of computational intelligence being applied to cancer-related detection.

This distinction is important when interpreting the historical development of AI in oncology. The emergence of computational approaches in cancer research did not necessarily coincide with the adoption of the term *artificial intelligence*. Pattern recognition, cybernetics, automated classification, expert systems, and other computational techniques developed under different conceptual and technological traditions. Some subsequently became closely associated with AI, whereas others maintained partly independent trajectories. Consequently, restricting a historical analysis exclusively to the expression *artificial intelligence* would risk overlooking some of the earliest computational approaches used to address cancer-related problems.

The convergence becomes explicit in the 1980s. Friedman and Frank’s 1983 study, *Use of conditional rule structure to automate clinical decision support: a comparison of artificial intelligence and deterministic programming techniques* [8], provides a particularly clear milestone for Position 5. The paper explicitly discusses artificial intelligence rule-based systems in the context of clinical decision support, oncology practice, and clinical research. Unlike the earlier 1967 record, in which the connection is established through pattern recognition and cybernetic classification, the 1983 publication explicitly frames the computational approach in terms of *artificial intelligence*. It therefore marks a transition from the use of AI-related computational techniques in cancer applications to the explicit recognition of AI as a technological approach relevant to oncology.

Taken together, the five search positions provide a compact historical framework for understanding this convergence. Position 1 identifies early terminology that can retrospectively be related to later AI concepts; Position 2 establishes the much older historical trajectory of cancer as a medical domain; and Position 3 marks the emergence of *artificial intelligence* as an explicit scientific concept. Position 4 then identifies an early intersection between cancer and broader AI-related computational approaches, represented by automated pattern recognition and cybernetic classification in cancer detection. Finally, Position 5 captures the point at which artificial intelligence itself was explicitly associated with oncology and clinical decision support.

This sequence suggests that the relationship between cancer and AI should not be attributed to a single technological breakthrough. Rather, it emerged from the gradual intersection of two independently developing domains. Cancer research progressively generated increasingly complex diagnostic, biological, imaging, and clinical problems, while computational science developed methods for pattern recognition, classification, knowledge representation, and decision support. The 1967 Cesar system illustrates an early computational bridge between these trajectories, whereas the 1983 study illustrates their explicit convergence under the terminology of artificial intelligence.

From this historical perspective, the subsequent expansion of machine learning, deep learning, computer vision, natural language processing, generative AI, foundation models, and multimodal systems can be viewed not as an entirely new phenomenon, but as successive stages in an already established process of computational integration. What has changed most dramatically is the scale, diversity, and complexity of the data available to oncology, together with the increasing capacity of AI systems to integrate information across research, diagnosis, prognosis, treatment, and patient care. This progressive transformation provides the historical context for the current development of intelligent approaches across the cancer continuum and for the scientific contributions collected in this Special Issue.

*Where Cancer Research Meets AI Today*

The historical convergence described above has developed into a broad and rapidly expanding research landscape in which artificial intelligence (AI) is being investigated across the cancer continuum. Current applications extend from cancer research and early detection to diagnosis, prognosis, treatment planning, clinical decision support, symptom monitoring, and survivorship. AI is therefore increasingly being considered not simply as a tool for data analysis, but as a computational framework for pattern recognition, prediction, classification, information integration, and support of increasingly individualized approaches to cancer care [9,10,11,12,13,14].

One of the most established areas of application is cancer detection and diagnosis through medical imaging and digital pathology. AI-based approaches have been investigated across a wide range of imaging modalities and cancer types, with applications including lesion detection, classification, segmentation, and diagnostic support [9]. Digital pathology represents a particularly active field. A systematic review and meta-analysis of AI applied to whole-slide images found high reported diagnostic accuracy across a broad range of diseases, while also identifying substantial concerns regarding risk of bias, applicability, validation, and reporting quality [10]. These findings illustrate both the potential of AI-assisted image interpretation and the importance of rigorous evaluation before widespread clinical implementation.

The role of AI is also expanding beyond image interpretation toward the integration of pathological, molecular, and clinical information. In oncology pathology, AI-based approaches have been investigated for tumour diagnosis, molecular biomarker detection, and prognosis assessment [11]. More recently, multimodal models combining pathology images with high-throughput omics have attracted increasing attention. Such approaches seek to integrate complementary biological and morphological information and may provide more comprehensive representations of tumour characteristics and patient outcomes. However, the available evidence also highlights important limitations concerning external validation, bias, reporting quality, and clinical applicability [12].

Prognosis and treatment-response prediction constitute another major area of AI research in oncology. Machine-learning approaches have been investigated for survival prediction, recurrence and progression assessment, risk stratification, and estimation of treatment outcomes. The increasing availability of multimodal datasets may allow these models to combine clinical, pathological, imaging, and molecular information rather than relying on a single data source [11,12]. This development is particularly relevant to precision oncology, where the objective is increasingly to characterize individual disease trajectories and identify patients who may benefit from different therapeutic strategies.

AI is similarly being explored in treatment planning and clinical decision support. Current applications include prediction of treatment response, support for therapeutic selection, and integration of complex clinical information. The potential value of these systems lies in their ability to process large and heterogeneous datasets and identify patterns that may complement clinical expertise. Nevertheless, predictive performance alone does not establish clinical utility. External validation, prospective evaluation, interpretability, workflow integration, and demonstration of benefit for patients remain essential requirements for meaningful clinical translation [11,12].

Another important area concerns cancer clinical trials. AI has been investigated to facilitate the identification of potentially eligible patients and improve the efficiency of trial recruitment. A systematic review and meta-analysis including more than 50,000 patients across 19 datasets found that AI-based approaches generally achieved high sensitivity and specificity for identifying patients potentially eligible for cancer clinical trials, although the authors emphasized the need for broader validation and attention to generalizability [13]. These approaches illustrate how AI may contribute not only to clinical care but also to the organization and acceleration of cancer research.

The application of AI is increasingly extending into longitudinal cancer care and survivorship. A systematic review of AI for symptom monitoring in adult cancer survivors identified 41 eligible studies from more than 18,000 records. Machine learning, natural language processing, AI-driven chatbots, and decision-support systems were among the approaches investigated, using textual information, patient-reported symptoms, and physiological measurements as important sources of data [14]. These findings suggest a growing role for AI in monitoring the patient beyond individual clinical encounters and in supporting more personalized and patient-centered models of survivorship care.

Overall, the current landscape suggests a progressive movement from task-specific AI applications toward increasingly integrated approaches to oncology. Imaging, pathology, molecular information, clinical records, treatment data, and patient-generated information can increasingly be considered as complementary components of a broader computational representation of the patient. At the same time, the available evidence demonstrates that technological progress must be accompanied by attention to data quality, representativeness, generalizability, reproducibility, interpretability, and clinical validation [9,10,11,12,13,14]. The future development of intelligent oncology will therefore depend not only on increasingly sophisticated algorithms, but also on their reliable and responsible integration into clinical and research environments.

*Emerging Hot Topics and Future Directions*

Beyond established applications, several emerging directions may further transform the relationship between AI and oncology. Digital twins represent one particularly promising frontier. By creating dynamic computational representations of individual patients, digital and virtual twins may integrate clinical, biological, imaging, and longitudinal information to support disease modelling, treatment simulation, and personalized decision-making. A scoping review published in *Cancers* identified applications of digital and virtual twins across cancer diagnosis, therapy, and monitoring, while also highlighting substantial challenges related to data integration, scalability, privacy, real-time data acquisition, and clinical validation [15]. More broadly, a recent scoping review of digital twins for health emphasized the potential contribution of AI and data science while noting that healthcare digital twins remain at an early stage of development [16].

Generative AI represents another rapidly developing area. Large language models and other generative approaches are increasingly being investigated in oncology for processing and integrating complex information, supporting clinical and research activities, and facilitating interactions with medical knowledge. Recent reviews describe potential applications across diagnosis, prognosis, treatment planning, patient management, and clinical research, while also emphasizing concerns related to reliability, hallucination, privacy, validation, and responsible clinical use [17,18]. The rapid development of these technologies therefore creates considerable opportunities, but their integration into oncology requires evidence that extends beyond technical demonstrations.

Multimodal AI may be particularly relevant to oncology because cancer characterization inherently depends on multiple information sources. Radiological images, digital pathology, molecular profiles, laboratory data, clinical narratives, and longitudinal patient information provide complementary perspectives on disease. Multimodal large language models and related architectures are being developed to combine different modalities within unified computational frameworks. A recent systematic review of medical multimodal large language models identified applications involving multiple forms of medical data while also highlighting persistent challenges related to data heterogeneity, model reliability, hallucination, computational requirements, and clinical evaluation [19].

Another important future direction concerns longitudinal and adaptive AI. Cancer is a dynamic process, and disease characteristics, treatment responses, symptoms, and patient needs may change over time. AI systems capable of incorporating sequential information may therefore support dynamic risk assessment, treatment monitoring, and adaptive decision-making. In parallel, the combination of AI with remote monitoring and patient-generated data may extend intelligent oncology beyond conventional hospital-based encounters. Current evidence in cancer survivorship already demonstrates the use of AI with patient-reported symptoms and physiological information, although further work is required to establish clinical effectiveness and sustainable implementation [14].

These developments point toward a transition from isolated AI applications toward increasingly predictive, multimodal, adaptive, and patient-centered forms of intelligent oncology. The central challenge is no longer simply whether AI can be applied to cancer, but how different forms of computational intelligence can be integrated responsibly across the cancer continuum—from biological research and early detection to diagnosis, prognosis, treatment, monitoring, and survivorship.

Beyond these emerging developments, the scope of AI in oncology is also expanding toward other areas of translational and clinical innovation. These include AI-supported biomarker discovery and molecular characterization, drug discovery and repurposing, optimization of treatment strategies, and increasingly sophisticated approaches to clinical decision support and patient monitoring [20]. A recent systematic review of AI applications across cancer care identified five major areas of application—*imaging/radiomics, genomics/biomarker discovery, drug discovery/repurposing, clinical decision support, and patient monitoring—highlighting the increasingly broad role of AI across the cancer continuum* [20]. At the same time, the review emphasized persistent challenges related to data quality, heterogeneity, interpretability, validation, and translation into clinical practice [20]. Together, these developments reinforce the view of AI not as a single technological solution, but as an evolving ecosystem of computational approaches capable of supporting different stages of cancer research, diagnosis, treatment, and care.

*A Special Issue at the Intersection of Artificial Intelligence and Cancer Research, Diagnosis, and Care*

Against this rapidly evolving background, the Special Issue “The Use of Artificial Intelligence in Cancer Research, Diagnosis, and Care” [21] provides an opportunity to bring together the different dimensions of the increasingly broad relationship between artificial intelligence and oncology. From the early development of computational approaches for the recognition and classification of malignant patterns, through the progressive integration of machine learning and deep learning into cancer imaging, digital pathology, molecular characterization, prognosis, treatment planning, and clinical decision support, AI has progressively extended its role across the cancer continuum. More recent developments, including digital twins, generative AI, multimodal systems, foundation models, AI-supported biomarker discovery, and increasingly adaptive approaches to patient monitoring and personalized care, are further expanding this landscape. The contributions to this Special Issue provide an opportunity to examine these developments from complementary perspectives, encompassing established applications as well as emerging technologies, while addressing the methodological, clinical, ethical, and translational challenges that accompany their implementation. In this context, the Special Issue offers a timely snapshot of a field undergoing rapid transformation, in which artificial intelligence is progressively moving from task-specific computational applications toward increasingly integrated, predictive, multimodal, and patient-centered approaches to cancer research, diagnosis, and care.

## Figures and Tables

**Table 1:** Used composite keys.

Position	Brief Description	PubMed Title/Abstract Search
1	Broad AI-related landscape—identifies early records containing terminology or computational approaches that can be retrospectively related to the broader development of artificial intelligence.	(“artificial intelligence”[Title/Abstract] OR “AI”[Title/Abstract] OR “machine learning”[Title/Abstract] OR “deep learning”[Title/Abstract] OR “neural network*”[Title/Abstract] OR “artificial neural network*”[Title/Abstract] OR “convolutional neural network*”[Title/Abstract] OR “recurrent neural network*”[Title/Abstract] OR “transformer*”[Title/Abstract] OR “foundation model*”[Title/Abstract] OR “large language model*”[Title/Abstract] OR “generative artificial intelligence”[Title/Abstract] OR “generative AI”[Title/Abstract] OR “generative model*”[Title/Abstract] OR “reinforcement learning”[Title/Abstract] OR “supervised learning”[Title/Abstract] OR “unsupervised learning”[Title/Abstract] OR “self-supervised learning”[Title/Abstract] OR “representation learning”[Title/Abstract] OR “computer vision”[Title/Abstract] OR “natural language processing”[Title/Abstract] OR “computational intelligence”[Title/Abstract] OR “expert system*”[Title/Abstract] OR “pattern recognition”[Title/Abstract])
2	Emergence of cancer and oncology—identifies early records explicitly referring to cancer, neoplasms, tumors, malignancies, oncology, and related terminology in the Title/Abstract.	(cancer[Title/Abstract] OR cancers[Title/Abstract] OR neoplasm[Title/Abstract] OR neoplasms[Title/Abstract] OR tumor[Title/Abstract] OR tumors[Title/Abstract] OR tumour[Title/Abstract] OR tumours[Title/Abstract] OR carcinoma[Title/Abstract] OR carcinomas[Title/Abstract] OR malignancy[Title/Abstract] OR malignancies[Title/Abstract] OR oncology[Title/Abstract] OR oncologic[Title/Abstract] OR oncological[Title/Abstract])
3	Emergence of AI as an explicit concept—identifies early records explicitly using the term artificial intelligence in the Title/Abstract.	“artificial intelligence”[Title/Abstract]
4	Cancer/oncology–AI-related convergence—identifies cancer and oncology records using the broader set of AI-related terms and computational approaches in the Title/Abstract.	((cancer[Title/Abstract] OR cancers[Title/Abstract] OR neoplasm[Title/Abstract] OR neoplasms[Title/Abstract] OR tumor[Title/Abstract] OR tumors[Title/Abstract] OR tumour[Title/Abstract] OR tumours[Title/Abstract] OR carcinoma[Title/Abstract] OR carcinomas[Title/Abstract] OR malignancy[Title/Abstract] OR malignancies[Title/Abstract] OR oncology[Title/Abstract] OR oncologic[Title/Abstract] OR oncological[Title/Abstract]) AND (“artificial intelligence”[Title/Abstract] OR “AI”[Title/Abstract] OR “machine learning”[Title/Abstract] OR “deep learning”[Title/Abstract] OR “neural network*”[Title/Abstract] OR “artificial neural network*”[Title/Abstract] OR “convolutional neural network*”[Title/Abstract] OR “recurrent neural network*”[Title/Abstract] OR “transformer*”[Title/Abstract] OR “foundation model*”[Title/Abstract] OR “large language model*”[Title/Abstract] OR “generative artificial intelligence”[Title/Abstract] OR “generative AI”[Title/Abstract] OR “generative model*”[Title/Abstract] OR “reinforcement learning”[Title/Abstract] OR “supervised learning”[Title/Abstract] OR “unsupervised learning”[Title/Abstract] OR “self-supervised learning”[Title/Abstract] OR “representation learning”[Title/Abstract] OR “computer vision”[Title/Abstract] OR “natural language processing”[Title/Abstract] OR “computational intelligence”[Title/Abstract] OR “expert system*”[Title/Abstract] OR “pattern recognition”[Title/Abstract]))
5	Explicit cancer/oncology–AI association—identifies records in which cancer/oncology terminology and the explicit term artificial intelligence occur together in the Title/Abstract.	((cancer[Title/Abstract] OR cancers[Title/Abstract] OR neoplasm[Title/Abstract] OR neoplasms[Title/Abstract] OR tumor[Title/Abstract] OR tumors[Title/Abstract] OR tumour[Title/Abstract] OR tumours[Title/Abstract] OR carcinoma[Title/Abstract] OR carcinomas[Title/Abstract] OR malignancy[Title/Abstract] OR malignancies[Title/Abstract] OR oncology[Title/Abstract] OR oncologic[Title/Abstract] OR oncological[Title/Abstract]) AND “artificial intelligence”[Title/Abstract])
